# Whole blood transcriptomic investigation identifies long non-coding RNAs as regulators in sepsis

**DOI:** 10.1186/s12967-020-02372-2

**Published:** 2020-05-29

**Authors:** Lixin Cheng, Chuanchuan Nan, Lin Kang, Ning Zhang, Sheng Liu, Huaisheng Chen, Chengying Hong, Youlian Chen, Zhen Liang, Xueyan Liu

**Affiliations:** 1 Department of Critical Care Medicine, Shenzhen People’s Hospital, The Second Clinical Medicine College of Jinan University, Shenzhen, China; 2 Shenzhen People’s Hospital, The Second Clinical Medicine College of Jinan University, Shenzhen, China

**Keywords:** Sepsis, lncRNA, Functional module, Gene coexpression, Survival analysis, Differential analysis

## Abstract

**Background:**

Sepsis is a fatal disease referring to the presence of a known or strongly suspected infection coupled with systemic and uncontrolled immune activation causing multiple organ failure. However, current knowledge of the role of lncRNAs in sepsis is still extremely limited.

**Methods:**

We performed an in silico investigation of the gene coexpression pattern for the patients response to all-cause sepsis in consecutive intensive care unit (ICU) admissions. Sepsis coexpression gene modules were identified using WGCNA and enrichment analysis. lncRNAs were determined as sepsis biomarkers based on the interactions among lncRNAs and the identified modules.

**Results:**

Twenty-three sepsis modules, including both differentially expressed modules and prognostic modules, were identified from the whole blood RNA expression profiling of sepsis patients. Five lncRNAs, FENDRR, MALAT1, TUG1, CRNDE, and ANCR, were detected as sepsis regulators based on the interactions among lncRNAs and the identified coexpression modules. Furthermore, we found that CRNDE and MALAT1 may act as miRNA sponges of sepsis related miRNAs to regulate the expression of sepsis modules. Ultimately, FENDRR, MALAT1, TUG1, and CRNDE were reannotated using three independent lncRNA expression datasets and validated as differentially expressed lncRNAs.

**Conclusion:**

The procedure facilitates the identification of prognostic biomarkers and novel therapeutic strategies of sepsis. Our findings highlight the importance of transcriptome modularity and regulatory lncRNAs in the progress of sepsis.

## Background

Sepsis refers to the presence of a known or strongly suspected infection coupled with systemic and uncontrolled immune activation causes multiple organ dysfunction with worldwide mortality among 17–26% [1, 2]. Common symptoms of sepsis contain fever, increased heart rate, increased breathing rate and confusion, while specific symptoms include a cough with pneumonia or painful urination with a kidney infection. Sepsis can progress to septic shock with dramatically dropped blood pressure leading to a much higher mortality of 40% [3]. However, sepsis is a complex heterogeneous disease implicating a variety of cellular processes and we can hardly identify reliable diagnostic and prognostic biomarkers for sepsis in clinical [4].

High-throughput gene expression analysis can detect tens of thousands of genes simultaneously, which provides vast opportunities to improve prognostic accuracy and address clinical questions that otherwise cannot be answered. Transcriptomic strategies have been adopted among numerous diseases to investigate differential expression analysis, coexpression pattern, survival analysis, prediction modeling, etc., leading to substantial advances in the identification of promising diagnostic biomarkers as well as clinical use and disease treatment [4–6]. *Poll* and his colleagues have utilized the high-throughput blood gene expression profiling to carry out the comparative analysis of the systemic response for sepsis patients diagnosed in distinct subgroups and endotypes, such as community-acquired and hospital-acquired pneumonia, bacterial sepsis and fungal sepsis, hyper-inflammatory and hypo-inflammatory, and critically ill patients in different platelet counts [7–10]. Gene expression signatures and candidate plasma proteins have been identified and characterized from critically ill patients with different subtypes of sepsis [11–13].

Also, biological network analysis coupled with functional module analysis have been commonly deployed in the domain of cancer study, to probe the tumor biogenesis and dysfunction in patients with cancer, which facilitated the pathway and mechanism studies that otherwise would be hardly discovered [14]. We previously designed a procedure SMILE for the identification of protein modules taking account of the subcellular localization of proteins [15, 16]. The resulting modules showed high correspondence with known modules and canonical pathways. Moreover, a computational framework was proposed to predict moonlighting lncRNAs by clustering the protein interaction network to determine modules with independent functions [17].

Long non-coding RNAs (lncRNAs) are a type of transcripts with more than 200 nucleotides that have low protein-coding potential, which function in a variety of cellular processes and usually serve as disease diagnostic and prognostic markers [17, 18]. Numerous studies have implicated the mutations and dysregulations of lncRNAs contribute to the development of immunity diseases and cancers [19–21]. Accumulating evidence has demonstrated lncRNAs playing roles as competing endogenous RNAs (ceRNAs) to determine the fate of gene transcripts in a variety of diseases [22, 23]. However, the role of lncRNAs in sepsis remains largely unknown, although sporadic works reported that organ failure in sepsis is associated with the expression change of lncRNAs in some tissues, i.e., liver, kidney, and skeletal muscle [24]. Thus, in this context, we need to find new lncRNA therapeutic targets and investigate their regulatory mechanisms in sepsis for the severely ill sepsis patients.

We comprehensively performed an in silico investigation of the gene coexpression pattern for the patients response to all-cause sepsis in consecutive intensive care unit (ICU) admissions. Sepsis can be caused by a broad range of pathogens, including viruses, bacteria, fungi, and parasites. We investigate sepsis in this study regardless of the source of infection. We identified diagnostic modules based on the whole blood RNA expression profiles of sepsis patients, and subsequently predicted sepsis associated lncRNAs on the basis of the interactions among lncRNAs and the identified coexpression modules. After that, we established five candidate lncRNA regulators of sepsis and investigated their regulatory mechanism through miRNAs playing in a competing endogenous RNA fashion. Ultimately, FENDRR, MALAT1, TUG1, and CRNDE were reannotated using three independent expression cohorts and validated as differentially expressed lncRNAs.

## Materials and methods

### Gene expression datasets and data preprocessing

Microarray dataset GSE65682 collected from the NCBI GEO database were used as the primary dataset in this study (Table 1) [25]. Raw array data preprocessing was performed using the *affy* package in the R environment [26]. The raw gene expression matrixes were normalized by the RMA method [27–29]. Illumina chip dataset GSE69528 was adopted as validation dataset. The data were also preprocessed using R, which included quantile normalization, flooring all intensities < 10 to 10, and log2 transformation. Only the common genes detected in both datasets remained for analysis. Average expression intensities were used when multiple probe sets mapped an individual gene symbol. The *genefilter* algorithm was used to filter genes with interindividual variability over 0.5 [30], resulting in 11,222 most variable genes to construct the sepsis coexpression network. To overcome multiple comparison, Benjamini–Hochberg adjusted probabilities were used to define significance throughout the paper [31]. Adjusted P-value of 0.01 was used in this study if not stated otherwise.

**Table 1:** Whole blood expression datasets

GSE Number	Tissue	Control	Sepsis	Platform
*mRNA expression:*				
GSE65682 [13]	Whole blood	42	522	Affymetrix Human Genome U219 Array
GSE69528 [49]	Whole blood	28	83	Illumina HumanHT-12 V4.0 expression BeadChip
*lncRNA expression:*				
GSE95233 [50]	Whole blood	22	51	Affymetrix Human Genome U133 Plus 2.0 Array
GSE57065 [51]	Whole blood	25	28	Affymetrix Human Genome U133 Plus 2.0 Array
GSE28750 [41]	Whole blood	20	10	Affymetrix Human Genome U133 Plus 2.0 Array

Series GSE65682 was analyzed using the Affymetrix HG-U129 platform, including 42 healthy samples and 760 patients admitted to the ICU with sepsis. 522 patients with sepsis among them were picked up for further analysis. We used the dataset GSE65682 as the core discovery dataset and the primary results were based on this dataset, because it has the largest size of whole blood septic samples of adults and a large number of the samples have clinical information. Series GSE69528 contains 83 sepsis and 28 healthy whole blood samples analyzed using Illumina Human HT-12 V4.0 expression BeadChip. This dataset was used for the validation of module identification, as it has the second largest size of adult whole blood sepsis samples.

### Coexpression network construction

The sepsis expression cohort was independently processed using the weighted gene coexpression network analysis (WGCNA) for both datasets [32, 33]. A coexpression matrix is build up firstly, which is an adjacent matrix measuring the Pearson Correlation Coefficient (PCC) of all gene pairs. Then, a power function *f(x) *=* x*^*b*^ is used to tune the weighted matrix or network to be scale-free. A common linear model that regressed the connectivity frequency on gene connectivity is used to assess the network scale-free degree, with the fitting index R^2^ close to 1 indicates perfect organized. *b* was set as 6 for both datasets to construct the scale-free networks (Additional file 1: Figure S1 and S2). Afterward, the weighted coexpression matrix is transformed into a topological overlap matrix (TOM), which is a classical algorithm considering both direct and indirect interactions of all the gene members in the network, resulting in biologically more meaningful modules. Modules with gene number over 20 were determined for further analysis.

### Differentially expressed genes and modules

To identify differentially expressed genes (DEGs) between sepsis and normal samples, gene expression data were analyzed by the two-tailed t-test with a threshold of 0.01 and log2 transformed absolute Fold Change (FC) value of 1. A module is defined as Differentially Expressed Module (DEM) if the module significantly overrepresents the DEGs. Similarly, a module is defined up-regulated (or down-regulated) DEM if the module significantly overrepresents the up-regulated (or down-regulated) DEGs. The statistical significance is assessed by the Hypergeometric test with p-value less than 0.01, which is defined as follows,

$$ p = 1 - \mathop \sum \limits_{i = 0}^{k - 1} \frac{{\left( {\begin{array}{*{20}c} i \\ x \\ \end{array} } \right)\left( {\begin{array}{*{20}c} {m - i} \\ {n - x} \\ \end{array} } \right)}}{{\left( {\begin{array}{*{20}c} m \\ n \\ \end{array} } \right)}} $$

where n is the network size or the total number of genes of the coexpression network, m is the module size, x is the number of DEGs, and i is the number of DEGs included in the module. The *clusterProfiler* package in R was adopted to perform the functional annotation of the identified DEGs and gene modules [34]. The Hypergeometric test was also used to measure the consistence of two modules. Two modules, one from the primary dataset while the other from the validation dataset, are considered reproducible or significantly overlapped when the hypergeometric test P value is less than 0.01.

### Survival associated modules

Principal component analysis (PCA) was used to evaluate whether gene modules are relevant to the clinical outcome of sepsis patients. For each module, the first principal component of its gene members is calculated as module eigengene (ME), which served as the most representative gene expression of all genes in a module [18]. It was used to risk-stratified the sepsis patients into two subgroups. Then, we examined the correlation between ME and patient overall outcome to compute module-trait relevance. A module is associated with a survival outcome if the correlation p-value is below 0.05. Kaplan–Meier survival curves were used for illustrating the result of survival analysis, in which ME is the risk score assessing the prognosis ability. For the 760 sepsis samples of the discovery dataset, only 479 of them having clinical information were utilized for survival analysis.

### ncRNA-module interaction

The interactions between lncRNAs and gene products were obtained from two databases, LncRNA2Target v2.0 [35] and RAID v2.0 [36]. LncRNA2Target v2.0 is a high-confidence resource containing the relationships between lncRNAs and their target genes. We only adopted the literature mining low-throughput interactions. RAID v2.0 is an online repository of RNA–protein interactions, including interactions between proteins and lncRNAs, circRNAs, pseudogenes, and miRNAs, and only the experimental lncRNA-protein interactions were applied in this study. Together, 1724 lncRNAs with 31,179 gene/protein targets were established for further analysis. We define a lncRNA as module regulator if the genes in the module significantly overrepresent the target genes of the lncRNA (p-value < 0.01, hypergeometric test). The same strategy was also adopted for the miRNA-module interaction, where the miRNA targets were obtained from mirCode [37], mirDB [38], and mirTarBase [39]. For competing endogenous RNA analysis, only the lncRNA-module pairs sharing at least one miRNA were determined as a lncRNA-miRNA-mRNA interaction. Additionally, we performed a literature search of the sepsis related miRNAs and collected 30 unique miRNAs as the sepsis diagnostic miRNAs (Additional file 2: Table S1).

### Workflow of sepsis lncRNA identification

**Fig. 1:**
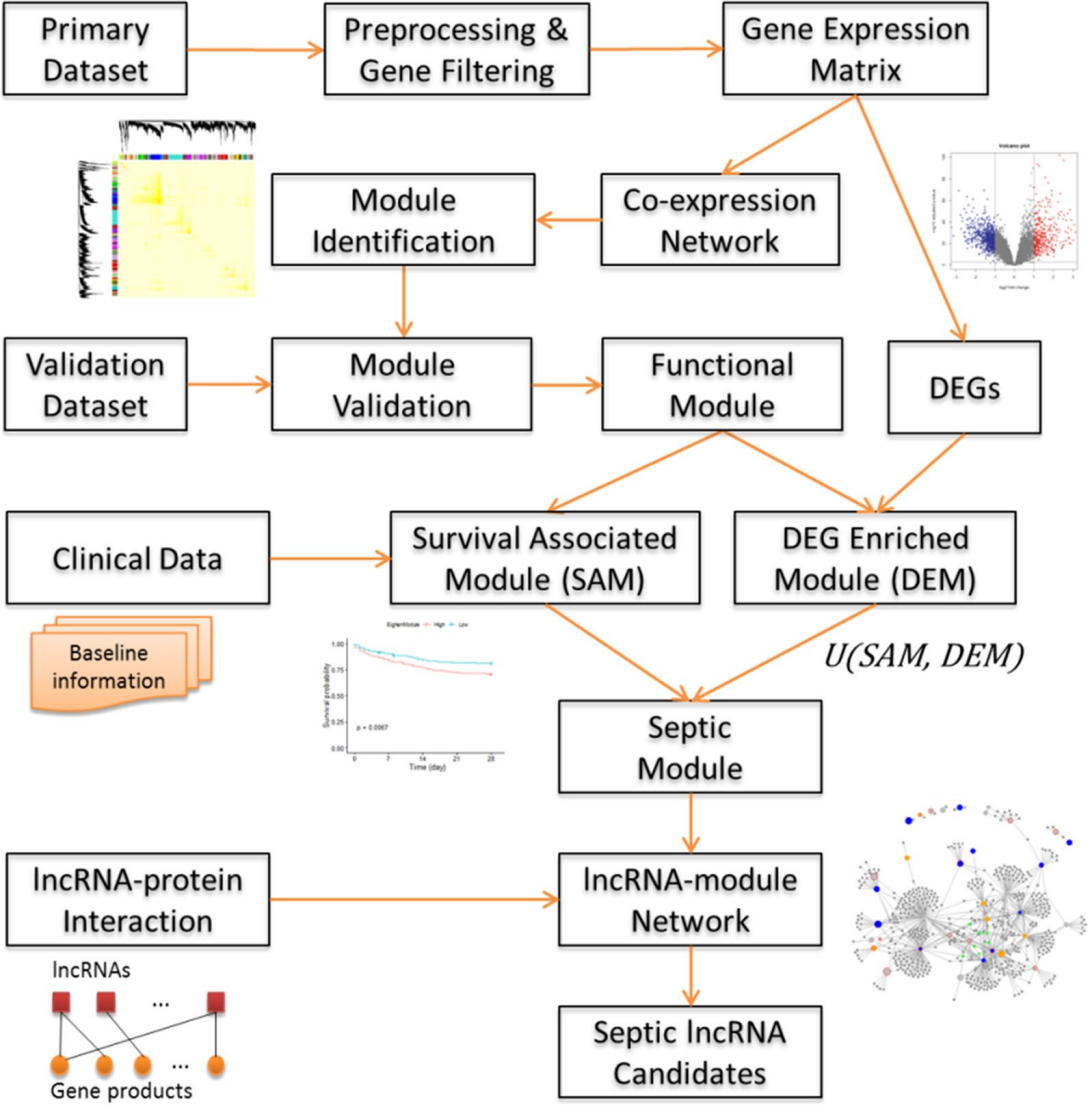
Workflow for the identification of sepsis lncRNA. DEGs, differentially expressed genes

As shown in Fig. 1, the main procedure consists of the 12 steps as follows:

1. Preprocess the raw data.
2. Establish the gene expression matrix for the genes with high variation.
3. Construct the gene coexpression network.
4. Identify gene coexpression modules using WGCNA.
5. Eliminate unstable modules by another expression dataset.
6. Screen differentially expressed genes (DEGs).
7. Identify modules enriched with DEGs (DEMs).
8. Calculate module eigengene and perform survival analysis.
9. Identify survival associated modules (SAMs).
10. Define sepsis modules by integrating DEMs and SAMs.
11. Construct the lncRNA-module interaction network.
12. Select sepsis candidate lncRNAs that are topologically critical.

We can obtain the sepsis lncRNA candidates using the procedure once the gene expression data, clinical data, and lncRNA-gene interaction data are imported.

### Reannotation of gene expression datasets

To explore how the lncRNAs are expressed in sepsis, we reannotated lncRNAs based on three sepsis adult whole blood gene expression datasets, GSE95233, GSE57065, and GSE28750. All of them are on the same platform of Affymetrix Human Genome U133 Plus 2.0 that were designed for detecting the expression intensity of coding genes. The platform of Affymetrix Human Genome U133 Plus 2.0 Array has been widely used for gene expression profiling of patient with sepsis [40, 41]. On top of this, it has the most comprehensive coverage of the annotated human lncRNAs. Using the latest NetAffx Annotation File, HG-U133_Plus_2 Annotations (Release 35, 04/16/15), we reannotated the lncRNAs of the three datasets as follows: (1) The RefSeq ID labeled with NR_ or XR_, indicative of non-coding RNAs, are retained; (2) the Ensemble gene IDs annotated with antisense, processed transcripts, sense overlapping, non-sense mediated decay, sense intronic or lincRNA are retained; (3) pseudogenes, rRNAs, microRNAs, and other small RNAs including tRNAs, snRNAs and snoRNAs are filtered out. Finally, 5016 probesets were detected as lncRNAs representing 3640 unique lncRNAs. Probesets encoding more than one lncRNA were averaged.

## Results

### Overview of workflow

We aimed to construct a lncRNA-module network composed of modules associated with sepsis pathology and lncRNAs with prognostic potential. To construct the network, we started by collecting sepsis gene expression datasets. Two datasets GSE65682 and GSE69528 were used in this study and were served as the primary and validation datasets, respectively. Then the analysis was performed mainly on the primary dataset following the procedure in Fig. 1. (1) Preprocessing the raw data using RMA. (2) Establishing the gene expression matrix for the genes with high expression variance. (3) Constructing the gene coexpression network represented by the Pearson correlation coefficients of all gene pairs. (4) Identifying gene coexpression modules using WGCNA. (5) Filtering out unstable modules by another validation expression dataset. Only the modules detected in both datasets were retained for subsequent analysis. (6) Screening DEGs between the sepsis and normal samples for the primary dataset. (7) Identifying DEMs using hypergeometric test. (8) Calculating module eigengene (ME) and perform survival analysis. (9) Identifying survival associated modules (SAMs) by examining the correlation between ME and patient survival outcome. (10) Combing DEMs and SAMs and define them as sepsis modules. (11) Constructing the lncRNA-module interaction network. The interactions were established using hypergeometric test to assess whether a sepsis module significantly overrepresents the target genes of a lncRNA. (12) Select the hub lnRNAs connecting more than three sepsis modules as sepsis candidate lncRNAs. Five sepsis lncRNAs were ultimately identified, FENDRR, MALAT1, TUG1, CRNDE, and ANCR.

### Coexpression network and modules

**Fig. 2:**
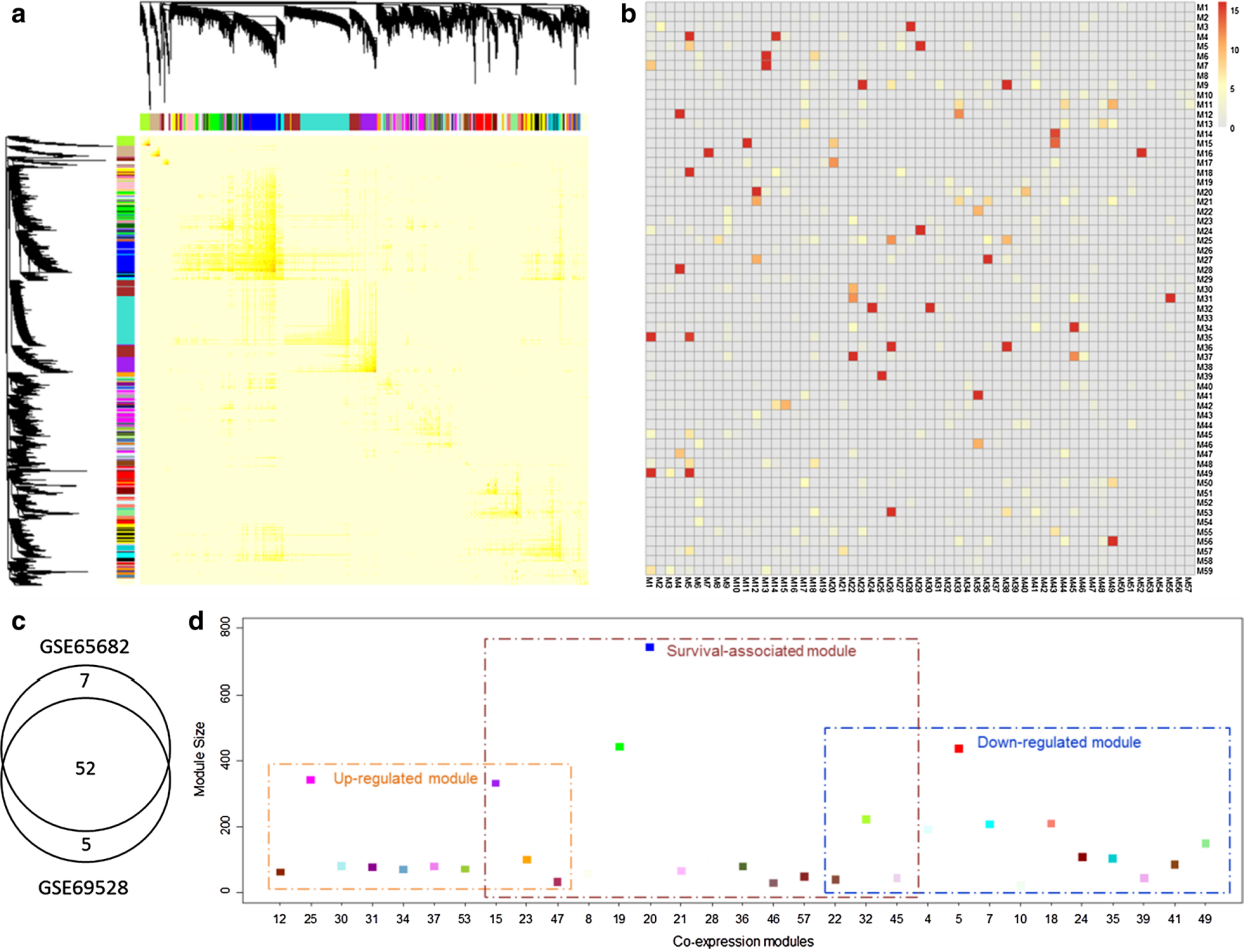
Module identification. **a** Identification of co-expression modules from the topological overlap matrix of GSE65682 using WGCNA. **b** Heatmap shows the reproducibility of two module lists. Rows represent modules identified from GSE65682 while columns represent modules from GSE69528. Grid color corresponds to –log10 transferred hypergeometric test P-values of the overlap of two modules. **c** Venn diagram of the identified modules from the two datasets. **d** Overview of the sepsis modules, including up-regulated DEMs, down-regulated DEMs, and SAMs. DEM, differentially expressed modules; SAMs, survival associated modules

The primary results were based on the GSE65682 dataset as it has the largest sample size. For this working dataset, we constructed a coexpression network consisting of 11,222 genes with expression variance over 0.5 across the sepsis patient samples. The topological overlap matrix illustrates an apparent organizational structure of the sepsis gene coexpression network, demonstrating that sepsis configures an array of specific coexpression structure. In total 59 modules were detected with sizes ranging from 30 to 750 (Fig. 2a). Different coexpression modules are highlighted in distinct colors. The detailed procedure of module identification and the module dendrogram are shown in Material and Method section and Additional file 1: Figure S1.

Using the same procedure, we also identified another set of gene module based on another independent microarray dataset GSE69528 for validation (Additional file 1: Figure S2 and S3). Common genes detected in both datasets were used for the coexpression network construction. Only the reproducible modules were retained for the subsequent analysis to investigate the expression change of modules during disease progression. As shown in Fig. 2b, rows are modules identified from our primary dataset GSE65682, while columns are modules determined from the validation dataset GSE69528. Significance of pairwise module overlap was measured by the -log10 transferred hypergeometric test p-values. It is clear that a high reproducibility was achieved for the two module lists. 52 out of 59 modules have at least one significant (P < 0.01, hypergeometric test) overlapping module in the validation dataset (Fig. 2c).

### Establishment of sepsis modules

**Fig. 3:**
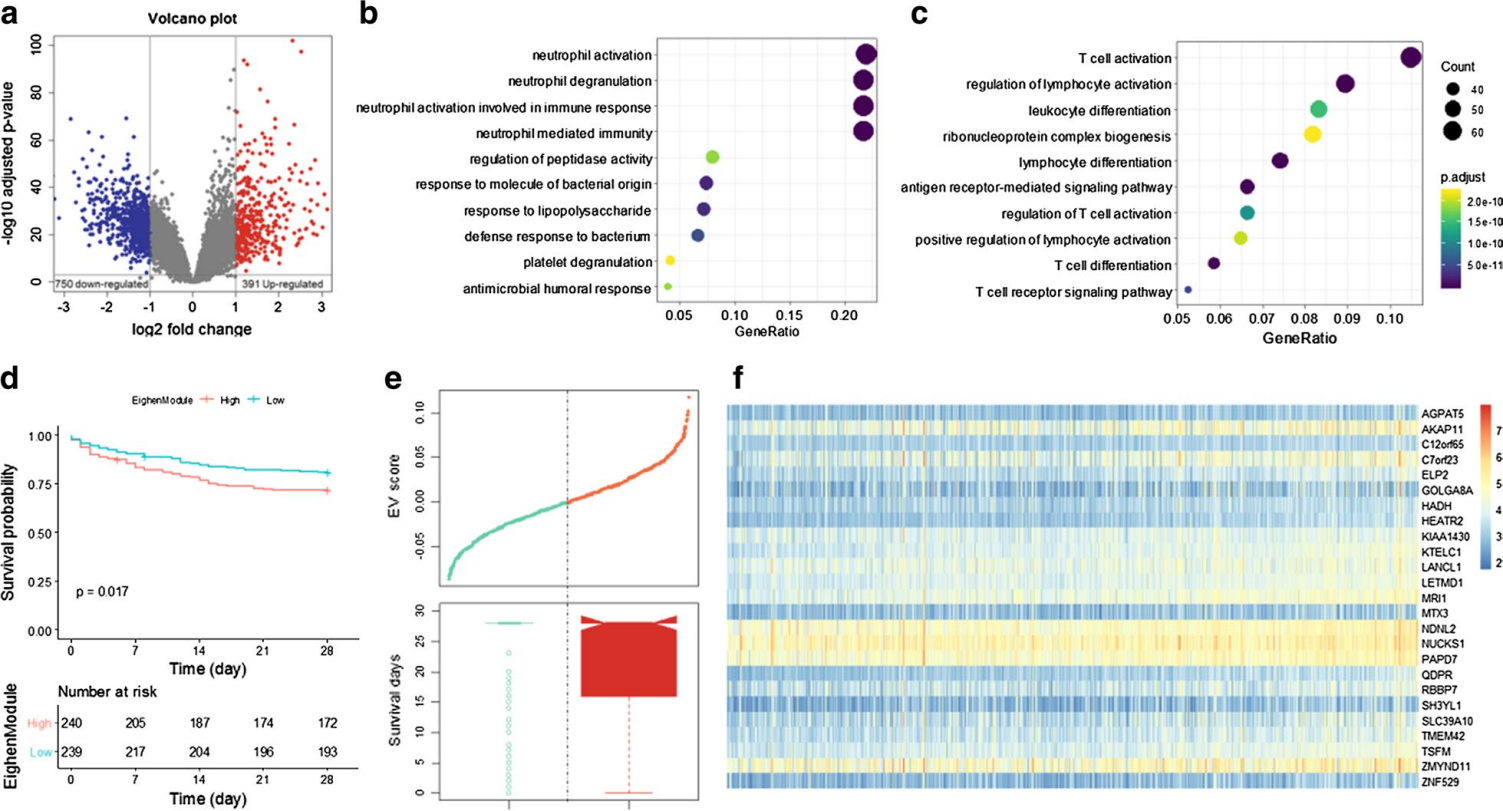
Sepsis module identification. **a** Volcano plot shows the differentially expressed genes. b, c) Functional analysis of the up- and down-regulated DEGs, respectively. **d** Kaplan–Meier curves of patients with sepsis at high (red) or low (light green) risk stratified by the EG scores of module 32. **e** Distribution of the EG scores (upper panel) and distributions of the survival time of two groups of patients (lower panel). The dotted line represents the median EG score dividing patients into two groups of high (red) and low risk (light green). **f** Heatmap of the expression profiles for the genes in module 32. Rows represent genes while columns represent patients

Using the t-test p-value of 0.01 and absolute fold change of 2 as thresholds, we screened 750 down-regulated DEGs and 391 up-regulated DEGs from the primary dataset (Fig. 3a). The down-regulated DEGs are significantly involved in biological processes like neutrophil mediated immunity, defense response to bacterium, platelet degranulation, etc. (Fig. 3b), while the up-regulated DEGs are enriched in the functional categories of T cell activation, Lymphocyte activation, T cell receptor signaling pathway, etc. (Fig. 3c).

To determine the expression difference of modules between the sepsis and normal samples, we adopted the hypergeometric test to evaluate whether a module significantly overrepresents up-regulated or down-regulated DEGs. A module is referred to as Differentially Expressed Module (DEM) if a substantial large fraction of genes is differentially expressed, indicating distinct expression pattern between the sepsis patients and the healthy samples. Thus, some modules are over expressed in sepsis whereas some others are low expressed. In total ten up-regulated and 13 down-regulated DEMs were detected from the sepsis coexpression network (Fig. 2d).

Moreover, to identify the modules associated with clinical outcome in sepsis, we performed multivariate Cox regression analysis to assess the significance of the correlation between patient overall survival and the Eigengene (EG) values of each module. As shown in Fig. 3e, the risk scores of the EG values were sorted with corresponding survival information for module 32. The dotted line in the middle of the figures corresponds to the median of EG value, which stratifies the sepsis patients into two subgroups with high and low risk. Figure 3d illustrates the Kaplan–Meier curves for the patients with clinical information according to the EG of M32. Patients with high EG values show much poorer prognostic values than those with low EG values, indicating that the dysfunction of M32 is close related to the prognosis of sepsis patients. The expression profiling of the DEGs in module 32 are illustrated as a heatmap in Fig. 3f. In total, we identified 14 modules from sepsis samples whose EGs are substantially correlated with patient overall survival and we defined them as survival-associated modules (SAM).

### Characteristic of sepsis modules

**Fig. 4:**
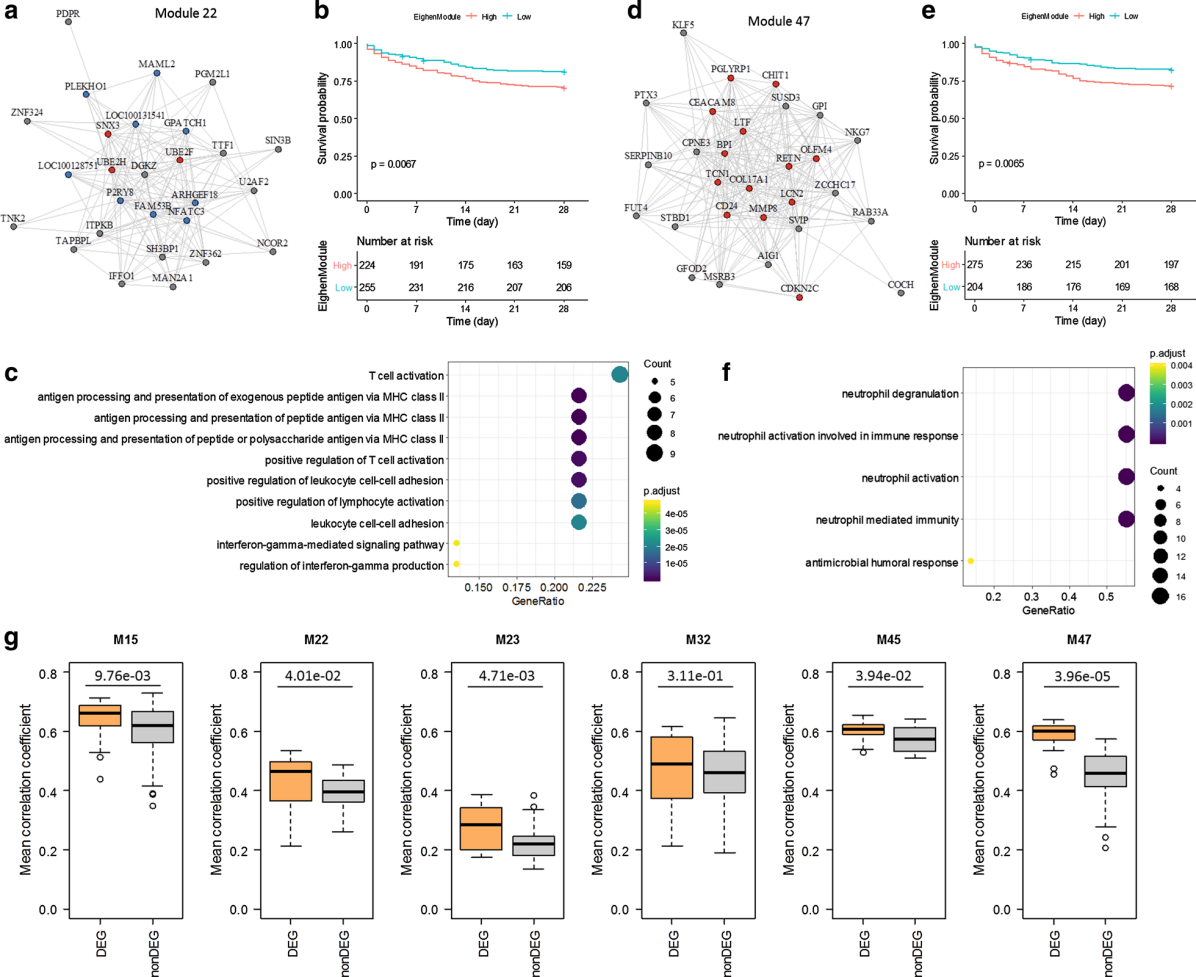
Identification of sepsis modules. **a** A co-expression module enriched of up-regulated DEGs. Vertexes correspond to genes and edges correspond to expression correlation. Only the edges with the absolute value of PCC greater than 0.5 are shown. Up-regulated DEGs are colored in red while down-regulated DEGs are colored in blue. **b** Kaplan–Meier curves of module 22. **c** Enriched GO biological processes of Module 22. **d** A co-expression module enriched of down-regulated DEGs. **e** Kaplan–Meier curves of module 47. **f** Enriched GO biological processes of Module 47. **g** The difference of mean correlation coefficient between DEGs and the other genes in different modules

31 sepsis modules, including both SAM and DEM, were screened from the primary dataset, implying novel gene signatures associated with sepsis pathology. We note an overlap of six modules (around 20%) between the two sets of SAM and DEM. Three of them are down-regulated, i.e., M22, M32, and M4, while the other three are up-regulated, i.e., M15, M23, and M47. The down-regulated module M22, for instance, consists of 22 genes closely co-expressed with each other; nine out of them are down-regulated DEGs playing as hub genes in the module (Fig. 4a). Kaplan–Meier curves were plotted for the rank-ordered Eigen Module values of M22 to carry out the 28-day survival analysis (Fig. 4b). It is apparent that patients with high EG value have substantial shorter survival time than those with low EG value. M22 are mainly implicated in biological processes like T cell activation, regulation of lymphocyte activation, leukocyte cell–cell adhesion, etc. (Fig. 4c). For the up-regulated module M47, it has 26 gene members and nine of them are DEGs up-regulated and more topologically important (Fig. 4d). The Kaplan–Meier curves show that patients with high EG value of M47 have a significantly worse prognosis than the low EG ones (Fig. 4e). M47 are involved in function categories of neutrophil mediated immunity as well as neutrophil activation and degranulation (Fig. 4f). Some other sepsis modules and their corresponding Kaplan–Meier curves are shown in Additional file 1: Figure S4 and S5.

Interestingly, we found that DEGs in the sepsis modules, either up-regulated or down-regulated, are prone to play a central role topologically in comparison to the non-DEGs. For instance, DEGs in M47 have an average correlation coefficient of 0.6 while the connectivity is merely 0.45 for the other genes (p < 3.96E−05, Mann–Whitney U test). Similar results can be observed for the other four modules of both SAM and DEM, including M15, M22, M23, and M45 (Fig. 4g). The DEGs of M32 are overall more correlated with module gene members in expression, although not significantly. Generally, the correlation coefficients of the DEGs is significantly higher than that of the non-DEGs in a module, suggesting that genes differentially expressed may drive the biogenesis or dysfunction of the coexpression gene modules.

### Sepsis lncRNA candidates

**Fig. 5:**
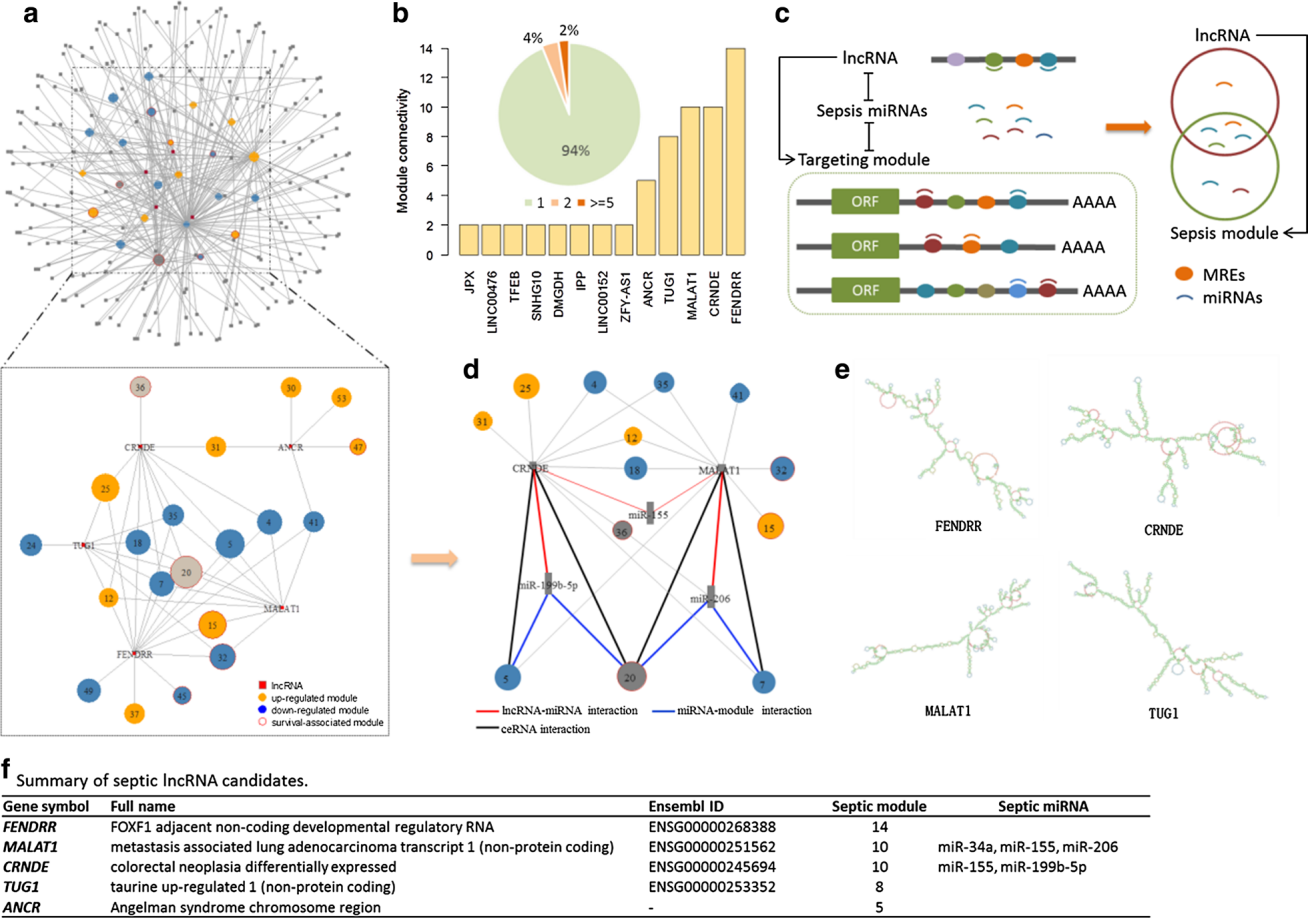
Sepsis candidate lncRNAs. **a** Overview of the lncRNA-module network. The bottom panel illustrate the subnetwork of sepsis candidate lncRNAs and interacting modules. Up-regulated DEMs are colored in red, down-regulated DEMs are colored in blue, and SAMs are framed in red circle. The node size of modules corresponds to module size. **b** Module connectivity of lncRNAs. The pie plot indicates the proportion of lncRNAs with different connectivity. The bar plot shows the lncRNAs linking more than one module. **c** ceRNA regulatory mechanism in sepsis. Short curves represent miRNAs and ellipses stand for MREs of lncRNAs or gene transcripts. The lncRNA sharing MREs with a gene module were hypothesized to regulate the module by competing for microRNA binding. **d** ceRNA interactions. Red and blue lines indicate lncRNA-miRNA and miRNA-module interactions, respectively. Bold black line represents the competing endogenous relationship. **e** Secondary structure of FENDRR, MALAT1, TUG1, and CRNDE. **f** Description of the sepsis lncRNA candidates. ceRNA, competing endogenous RNA. MREs, miRNA response elements. DEMs, differentially expressed modules. SAMs, survival associated modules

We constructed a lncRNA-module interaction network including 251 interactions between 23 sepsis modules and 201 lncRNAs (Fig. 5a). Although most of the lncRNAs regulate none or merely a single sepsis module (Fig. 5b), FENDRR, MALAT1, TUG1, CRNDE, and ANCR connect multiple sepsis modules with the connectivity of 14, 10, 10, 8, and 5, respectively, which are expected to have high potentials to be involved in the sepsis progress (Additional file 3).

A subnetwork concentrating on the five sepsis candidate lncRNAs and their regulated sepsis modules is shown on the bottom panel of Fig. 5a. FENDRR, the FOXF1 adjacent non-coding developmental regulatory RNA, plays as a hub regulator mediating 14 sepsis modules in the lncRNA-module interaction network. Both MALAT1 and CRNDE regulate ten sepsis modules and they share seven common modules, six out of them are down-regulated. TUG1 interacts with six down-regulated and two up-regulated DEMs, indicating that TUG1 tend to involve in under expression pathways. In contrast, ANCR (Angelman syndrome chromosome region) links four up-regulated DEMs and only one down-regulated DEMs, suggesting that ANCR may mediate some over expression pathways implicated in sepsis.

Furthermore, we investigated the regulatory mechanism of how the lncRNAs regulate the modules in sepsis from the perspective of competing endogenous RNAs (ceRNAs), which impact the translation rate of mRNAs by competing for shared miRNAs [22, 23]. lncRNAs are able to share the same miRNA response elements with mRNAs transferred by the sepsis modules, thereby sponging miRNAs intended to bind to these mRNAs and depressing the overall expression level of sepsis modules (Fig. 5c). Several miRNAs have been previously validated as potential regulators in sepsis, such as miR-34a, miR-206, and miR-199b-5p [42]. By these miRNAs, we found that CRNDE regulates module 5 and module 20 through miR-199b-5p (CRNDE ⟶ miR-199b-5p⟶ M5/M20), indicating that CRNDE acts as a miRNA sponge of miR-199b-5p and thereby modulating the transcripts of genes in module 5 and module 20 (Fig. 5d). Similarly, MALAT1 regulates module 7 and module 20 through the miR-206-mediated lncRNA-mRNA interactions (MALAT1 ⟶ miR-206 ⟶ M7/M20). The in-detail information of these candidate lncRNAs including secondary structure and miRNA targets are provided in Fig. 5e, f.

### Expression pattern of candidate lncRNAs

**Fig. 6:**
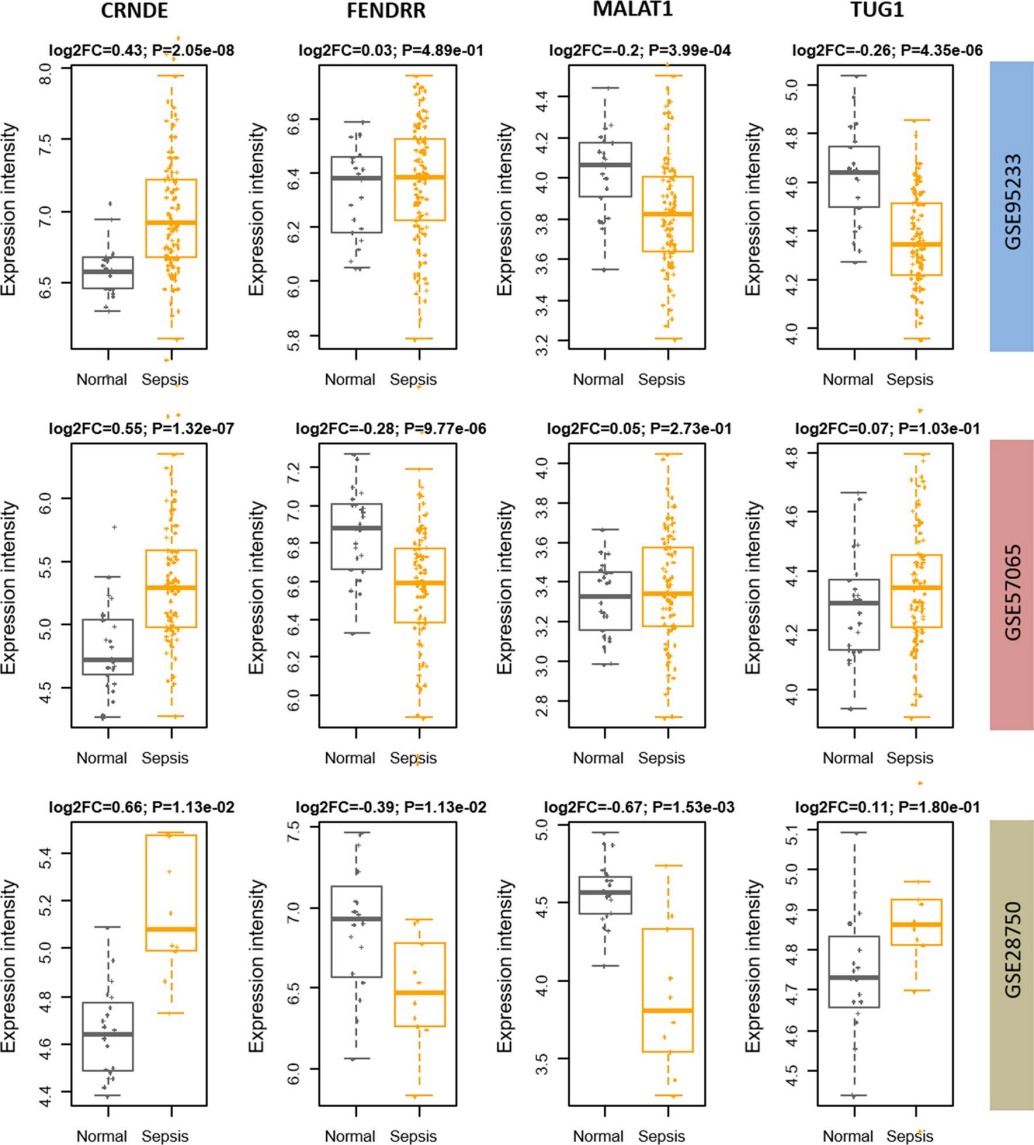
Validation of the sepsis candidate lncRNAs in three independent datasets. The four sepsis candidate lncRNAs, CRNDE, FENDRR, MALAT1 and TUG1, were detected as differentially expressed in at least one dataset

Following an lncRNA reannotation pipeline (see Methods), we reannotated the probes from three array datasets to obtain the lncRNA expression profiling. The gene coverages among distinct platforms are different. The platform Affymetrix HG-U133 Plus 2.0 Array detects much more genes than Affymetrix HG-U219 Array and the Illumina one. In other words, GSE65682 and GSE69528 contain quite limited number of lncRNAs for further differential analysis. After reannotation, we separately screened the differentially expressed lncRNAs (DELs) from the datasets of GSE95233, GSE57065, and GSE28750. Our finding shows that four out of the five sepsis lncRNA candidates are differentially expressed in at least one independent dataset except for ANCR, whose probes were not covered by the array platform (Fig. 6). Specifically, CRNDE is significantly up-regulated in the sepsis samples of all the three datasets, in which the (log2 transferred) fold changes are 0.43, 0.55, and 0.66, respectively. FENDRR and MALAT1 are significantly down-regulated in two datasets, while TUG1 is differentially expressed only in the dataset of GSE95233.

CRNDE, an oncogene that is usually overexpressed in tumor cells, contributes a lot to cellular proliferation, migration, invasion, and apoptosis [43]. More importantly, CRNDE can modulate the TLR3/NF-κB cytokine signaling pathway to trigger inflammation [44, 45], suggesting that CRNDE may serve as a regulator in sepsis. In sepsis, genes or gene modules inducted by MALAT1 may modulate their expression pattern in endothelial cells, which is critical as MALAT1 has been reported to mediate inflammation in traumatic brain injury [45]. Also, it was reported that TUG1 is able to affect the development of sepsis-associated acute kidney injury via modulating NF-κB pathway [46]. FENDRR has never been mentioned in the induction or progress of sepsis before, so it can be considered as a novel lncRNA regulator for sepsis.

## Discussion

We used a module-centric algorithm to identify sepsis lncRNAs via a network linking lncRNAs and coexpression modules. Twenty-three sepsis modules, including both differentially expressed modules and prognostic modules, were detected from the sepsis whole blood gene expression profiling. We identified five sepsis lncRNAs, FENDRR, MALAT1, TUG1, CRNDE, and ANCR, all of which connect five or more sepsis modules, indicating their functions are highly related with biological processes of sepsis. Further, we probed the regulatory mechanism of CRNDE and MALAT1 which act as competing endogenous RNAs (ceRNAs). CRNDE interacts with module 5 and module 20 through miR-199b-5p, while MALAT1 sponges miR-206 to regulate the target module 7 and module 20. At last, the five sepsis lncRNAs were independently validated in three gene expression datasets of sepsis. Four out of them were reannotated and detected as differentially expressed lncRNAs in at least one dataset.

Genome-wide expression study of sepsis is relatively at its infancy and several technologies prevalently used in other diseases have not been widely adopted in sepsis. In order to detect the sepsis lncRNAs, we integrated the conventional approaches including gene coexpression, module identification, differential analysis, survival analysis, and lncRNA-gene interaction, as well as mathematical and statistical algorithms. We comprehensively studied the gene coexpression pattern of patients with all-cause sepsis in ICU admissions in this study, although sepsis is a heterogeneous immunity disease and the mortalities of sepsis patients in distinct subtypes are substantially different [47]. In the future, we will investigate the coexpression pattern of patients with sepsis in specific subtypes, such as community-acquired and hospital-acquired pneumonia, bacterial sepsis and fungal sepsis, hyper-inflammatory and hypo-inflammatory, and endotypes classified by platelet counts [7, 9].

Since the interactions among lncRNAs and target genes are far from complete, the discovery of sepsis lncRNAs is limited by the interaction coverage [17, 18]. An alternative strategy is to produce the genome-wide RNA-seq data including both coding and non-coding genes, then a coding-non-coding network can be constructed and the association among coding and non-coding genes would be well established [48]. Undoubtedly, co-expression correlation is a key characteristic in gene function studies, although it is often biased due to the small simple size and the disproportionately large contributions of a fraction of samples. To improve the reliability, we will use the combination of protein–protein interactions and gene co-expression correlations to identify gene modules that are active in sepsis samples in future studies.

This is the first work computationally detecting the sepsis lncRNAs using coexpression and network analysis for application in the intensive care unit environment. Also, FENDRR is first proposed as a sepsis related lncRNA. The predicted sepsis lncRNAs is helpful for the diagnosis of sepsis and can improve our understanding of sepsis progress and development, although further experimental validation is required to elaborate how lncRNAs modulate the molecular signaling pathways of sepsis. The procedure will facilitate the identification of other types of sepsis-related molecules, such as circRNAs and pseudogenes, for the patients in critical care settings.

## Conclusion

This study identified five lncRNAs as sepsis regulators based on the interactions among lncRNAs and the identified sepsis modules, four of which were differentially expressed in three independent datasets. The procedure facilitates the identification of prognostic biomarkers and novel therapeutic strategies of sepsis. Our findings highlight the importance of transcriptome modularity and regulatory lncRNAs in the progress of sepsis.

## Supplementary information

**Additional file 1: Figure S1.** Identification of co-expression modules for dataset GSE65682. A) Parameter setup. B) Gene dendrogram and module colors. C) Module dendrogram. **Figure S2.** Identification of co-expression modules for dataset GSE69528. A) Parameter setup. B) Gene dendrogram and module colors. C) Module dendrogram. **Figure S3.** Identification of co-expression modules from the topological overlap matrix using WGCNA for dataset GSE69528. **Figure S4.** Kaplan–Meier curves of two patient groups with higher or lower EG value for module 15, 23, 45, and 36, respectively. **Figure S5.** Example of the coexpression modules enriched of up (31 and 37) or down-regulated DEGs (45 and 39). Vertexes correspond to genes and edges correspond to expression correlation. Only the edges with the absolute value of PCC greater than 0.5 are shown. Up-regulated DEGs are colored in red while down-regulated DEGs are in blue.

**Additional file 2:** Supplementary table of the curated sepsis miRNA biomarkers.

**Additional file 3:** Survival-associated modules.

## Data Availability

Data are available on request.
