# Supplementary material for: Whole blood transcriptomic investigation identifies long non-coding RNAs as regulators in sepsis
Source: J Transl Med. 2020 May 29;18:217. doi: 10.1186/s12967-020-02372-2 (PMC7257169; doi:10.1186/s12967-020-02372-2)
Supplement: Supplementary file 1 — Additional file 1: Figure S1. Identification of co-expression modules for dataset GSE65682. A) Parameter setup. B) Gene dendrogram and module colors. C) Module dendrogram. Figure S2. Identification of co-expression modules for dataset GSE69528. A) Parameter setup. B) Gene dendrogram and module colors. C) Module dendrogram. Figure S3. Identification of co-expression modules from the topological overlap matrix using WGCNA for dataset GSE69528. Figure S4. Kaplan–Meier curves of two patient groups with higher or lower EG value for module 15, 23, 45, and 36, respectively. Figure S5. Example of the coexpression modules enriched of up (31 and 37) or down-regulated DEGs (45 and 39). Vertexes correspond to genes and edges correspond to expression correlation. Only the edges with the absolute value of PCC greater than 0.5 are shown. Up-regulated DEGs are colored in red while down-regulated DEGs are in blue. [file 12967_2020_2372_MOESM1_ESM.docx]

**Whole blood transcriptomic investigation identifies prognostic gene modules and long non-coding RNAs in sepsis**


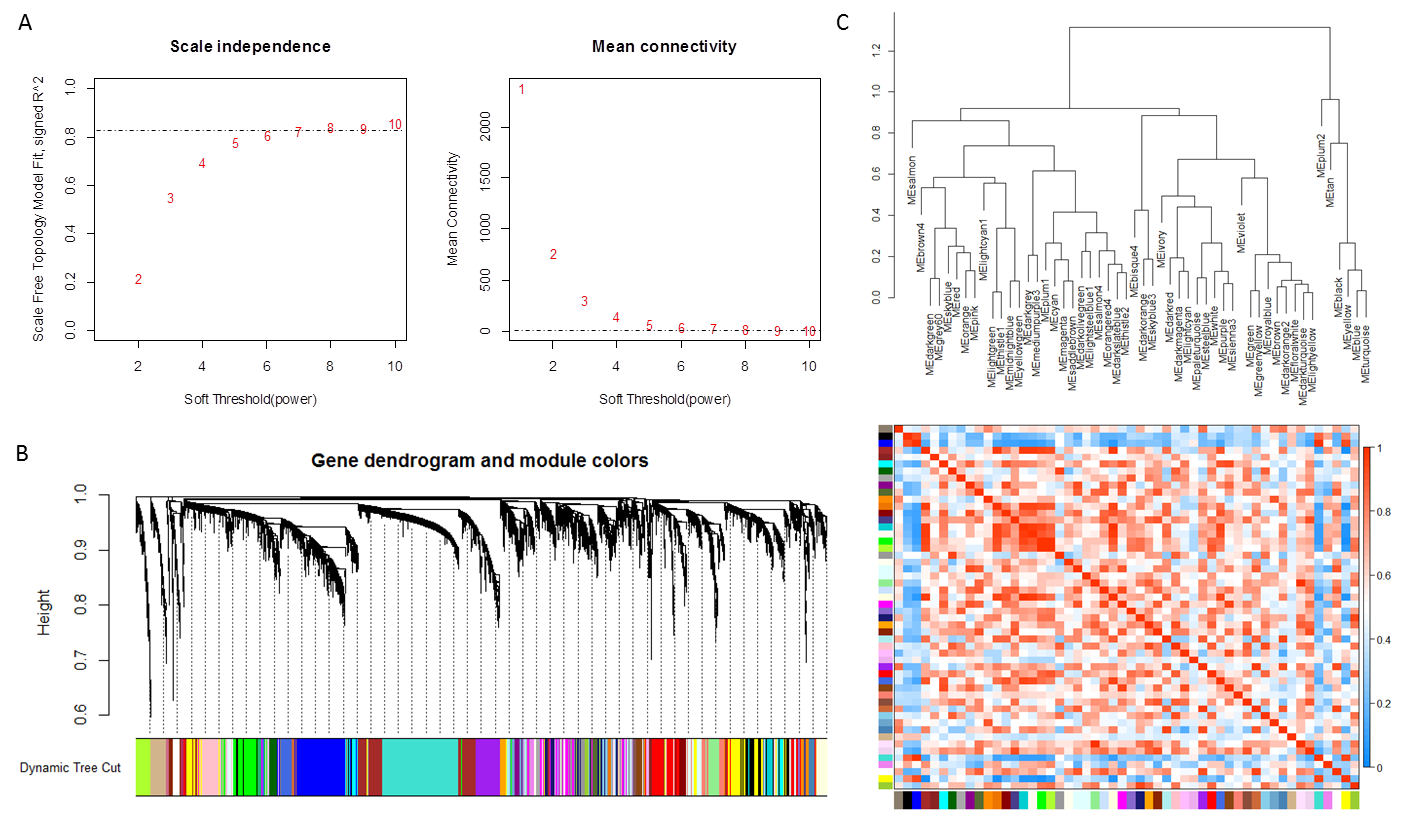


Figure S1. Identification of co-expression modules for dataset GSE65682. A) Parameter setup. B) Gene dendrogram and module colors. C) Module dendrogram.


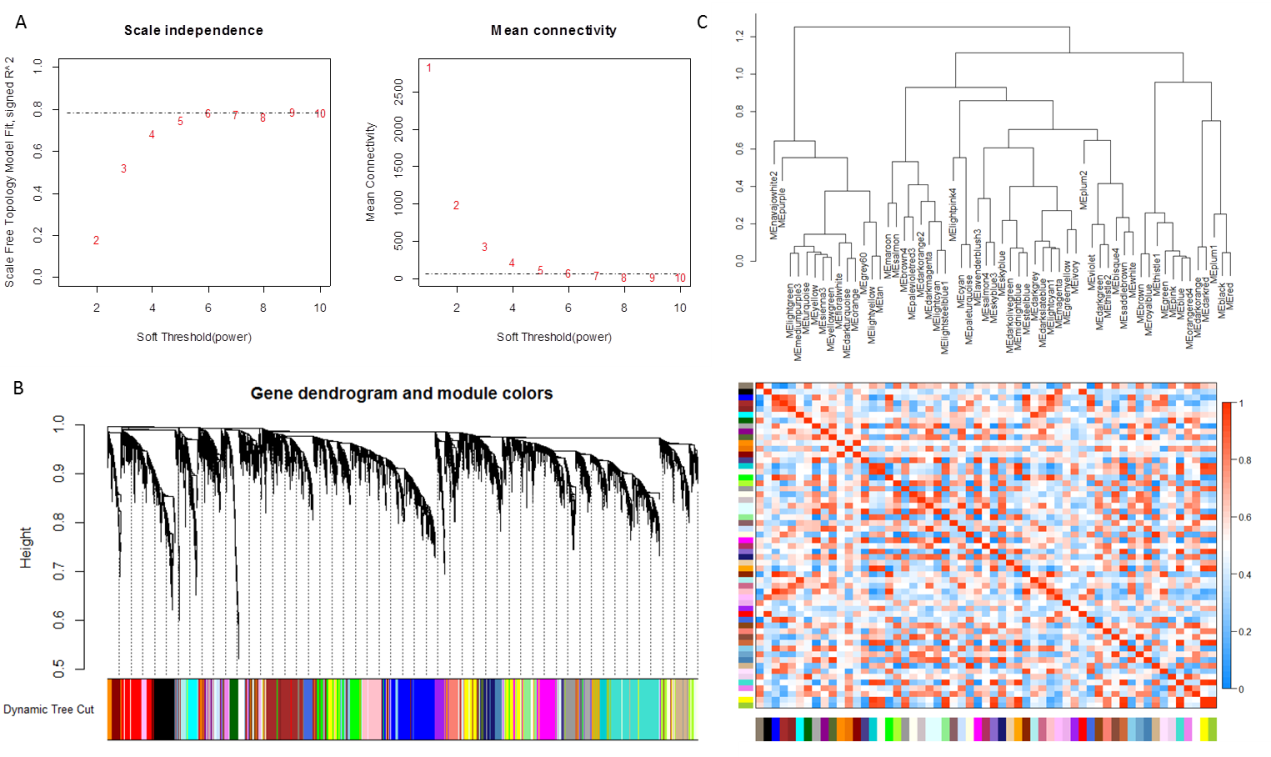


Figure S2. Identification of co-expression modules for dataset GSE69528. A) Parameter setup. B) Gene dendrogram and module colors. C) Module dendrogram.


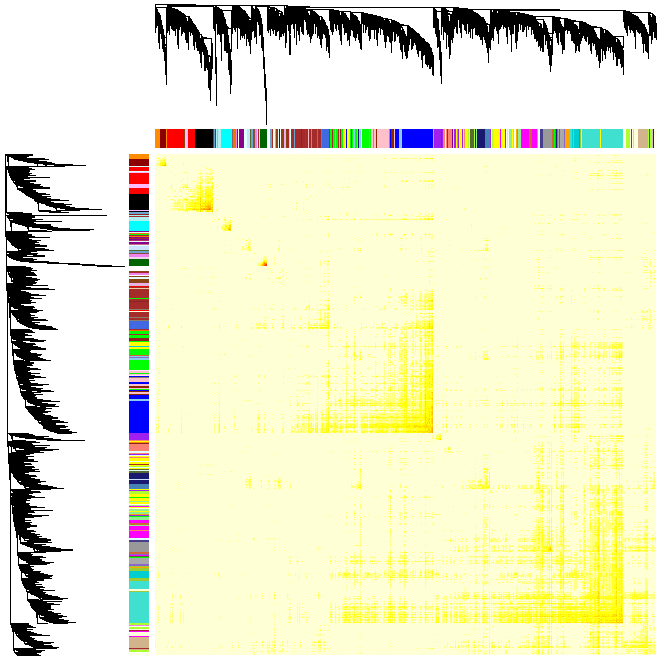


Figure S3. Identification of co-expression modules from the topological overlap matrix using WGCNA for dataset GSE69528.


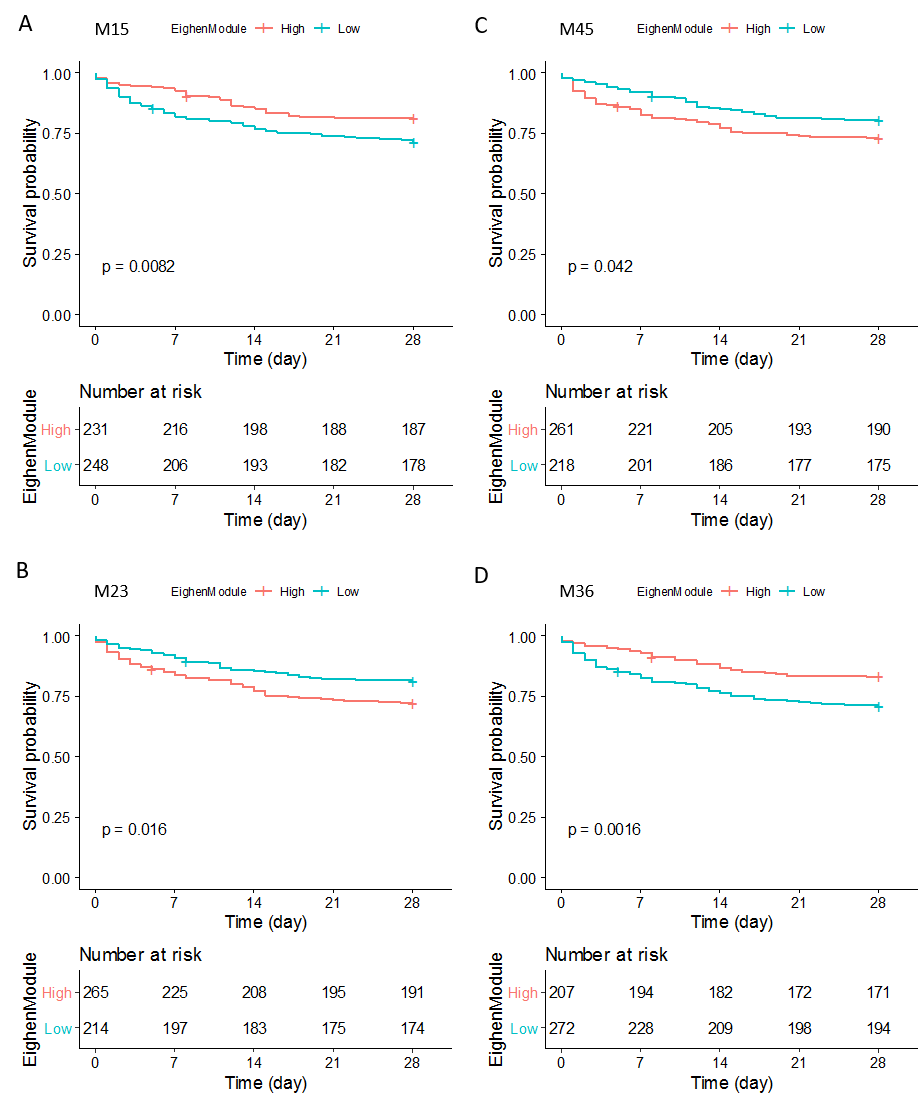


Figure S4. Kaplan–Meier curves of two patient groups with higher or lower EG value for module 15, 23, 45, and 36, respectively.


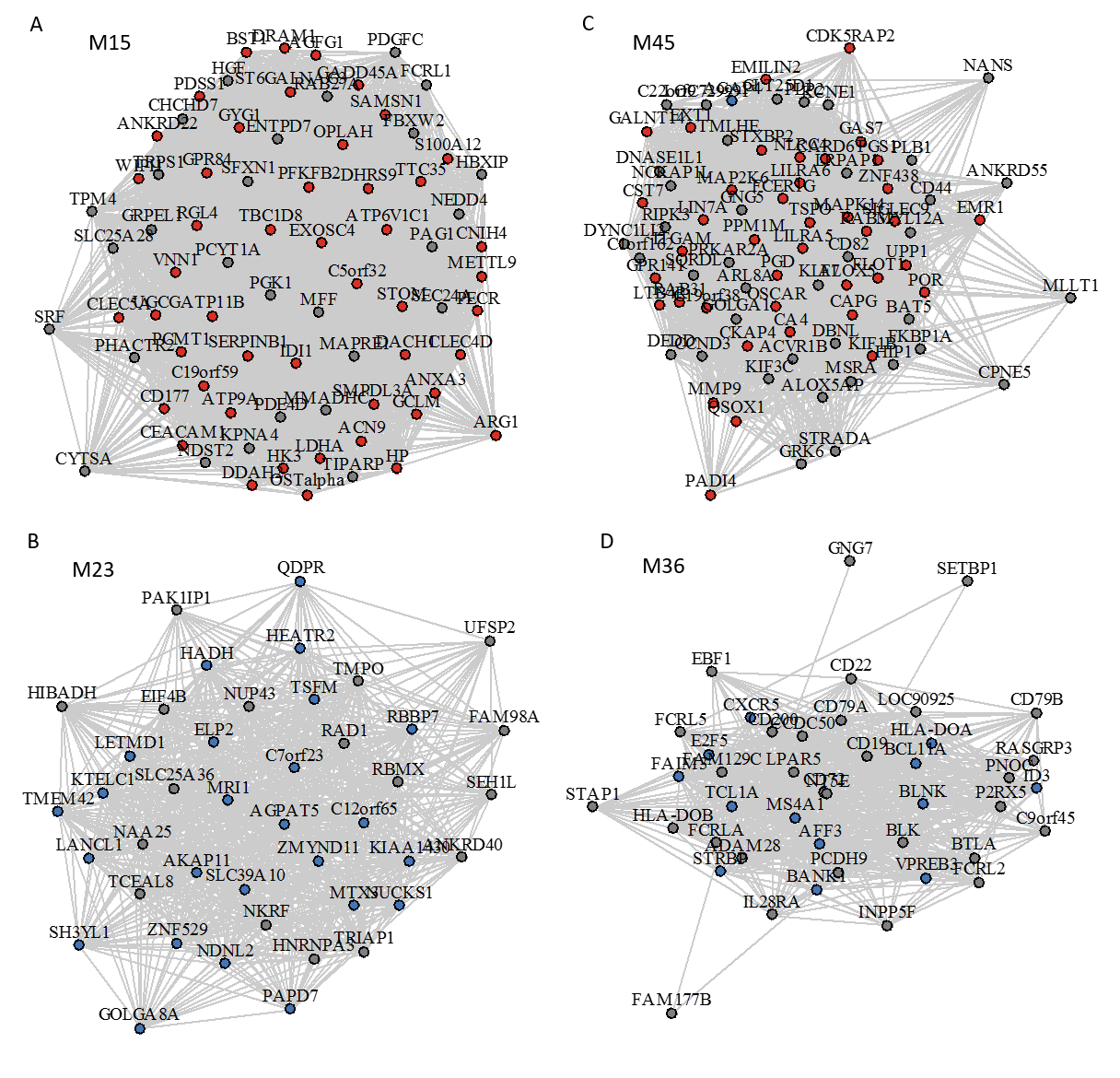


Figure S5. Example of the coexpression modules enriched of up (31 and 37) or down-regulated DEGs (45 and 39). Vertexes correspond to genes and edges correspond to expression correlation. Only the edges with the absolute value of PCC greater than 0.5 are shown. Up-regulated DEGs are colored in red while down-regulated DEGs are in blue.
